# HIV Lipodystrophy in Participants Randomised to Lopinavir/Ritonavir (LPV/r) +2–3 Nucleoside/Nucleotide Reverse Transcriptase Inhibitors (N(t)RTI) or LPV/r + Raltegravir as Second-Line Antiretroviral Therapy

**DOI:** 10.1371/journal.pone.0077138

**Published:** 2013-10-30

**Authors:** Allison Martin, Cecilia L. Moore, Patrick W. G. Mallon, Jennifer F. Hoy, Sean Emery, Waldo H. Belloso, Praphan Phanuphak, Samuel Ferret, David A. Cooper, Mark A. Boyd, on behalf of the Second-line study team

**Affiliations:** 1 The Kirby Institute, University of New South Wales, Sydney, New South Wales, Australia; 2 University College Dublin School of Medicine and Medical Science, Dublin, Ireland; 3 The Alfred Hospital and Monash University, Victoria, Melbourne, Australia; 4 Coordinación de Investigación Clínica Académica en Latinoamérica, Buenos Aires, Argentina; 5 Thai Red Cross AIDS Research Center, Bangkok, Thailand; 6 Hopital Saint-Louis, Paris, France; Asociacion Civil Impacta Salud y Educacion, Peru

## Abstract

**Objective:**

To compare changes over 48 weeks in body fat, lipids, Metabolic Syndrome and cardiovascular disease risk between patients randomised 1∶1 to lopinavir/ritonavir (r/LPV) plus raltegravir (RAL) compared to r/LPV plus 2–3 nucleoside/nucleotide reverse transcriptase inhibitors (N(t)RTIs) as second-line therapy.

**Methods:**

Participants were HIV-1 positive (>16 years) failing first-line treatment (2 consecutive HIV RNA >500 copies/mL) of NNRTI +2N(t)RTI. Whole body dual energy x-ray absorptiometry was performed at baseline and week 48. Data were obtained to calculate the Metabolic Syndrome and Framingham cardiovascular disease (CVD) risk score. Linear regression was used to compare mean differences between arms. Logistic regression compared incidence of metabolic syndrome. Associations between percent limb fat changes at 48 weeks with baseline variables were assessed by backward stepwise multivariate linear regression. Analyses were adjusted for gender, body mass index and smoking status.

**Results:**

210 participants were randomised. The mean (95% CI) increase in limb fat over 48 weeks was 15.7% (5.3, 25.9) or 0.9 kg (0.2, 1.5) in the r/LPV+N(t)RTI arm and 21.1% (11.1, 31,1) or 1.3 kg (0.7, 1.9) in the r/LPV+RAL arm, with no significant difference between treatment arms (−5.4% [−0.4 kg], p>0.1). Increases in total body fat mass (kg) and trunk fat mass (kg) were also similar between groups. Total:HDL cholesterol ratio was significantly higher in the RAL arm (mean difference −0.4 (1.4); p = 0.03), there were no other differences in lipid parameters between treatment arms. There were no statistically significant differences in CVD risk or incidence of Metabolic Syndrome between the two treatment arms. The baseline predictors of increased limb fat were high viral load, high insulin and participant's not taking lipid lowering treatment.

**Conclusion:**

In patients switching to second line therapy, r/LPV combined with RAL demonstrated similar improvements in limb fat as an N(t)RTI + r/LPV regimen, but a worse total:HDL cholesterol ratio over 48 weeks.

**Trial Registration:**

This clinical trial is registered on Clinicaltrials.gov, registry number NCT00931463.

## Introduction

HIV associated lipodystrophy is a syndrome of peripheral lipoatrophy, central fat accumulation, and lipid derangement. Lipodystrophy complicates the management of HIV-infected patients through dyslipidaemia, increased cardiovascular disease (CVD) risk and cosmetic affect. Both HIV infection itself and long term exposure to combination antiretroviral therapy (cART) have been implicated in the pathogenesis of lipodystrophy, which can affect up to 50% of individuals receiving cART [1]–[4]. The use of thymidine analogue nucleotide reverse transcriptase inhibitors (ta-NRTIs) has been minimised in high-income countries, as they have been implicated as the main cause of lipoatrophy and other severe adverse events [1]–[8]. However, ta-NRTIs are still commonly used as first-line treatment in low and middle-income countries because of their comparatively low cost.

Changes in circulating lipoproteins have been demonstrated with use of three of the major antiretroviral drug classes (protease inhibitors [PI], nucleoside/nucleotide reverse transcriptase inhibitors [N(t)RTI] and non-nucleoside reverse transcriptase inhibitors [NNRTI]), although the pattern of changes differ between and among the three drug classes [5], [9]–[13]. Recent clinical trials using the integrase inhibitor, raltegravir (RAL), in antiretroviral naïve [14], [15] and cART experienced participants [16], [17] have reported various effects on lipids. Results vary from reports of small increases [14] to significant increases [15], [16], whereas others report improvements [17] in the lipid profile, compared to N(t)RTIs, PIs or efavirenz. An *in-vitro* study has demonstrated RAL had minimal affects on the expression of peroxisome proliferator activated receptor (PPAR-γ) and sterol regulatory element binding protein (SREBP-1c), which are involved in lipid accumulation [18]. Adipose tissue changes associated with RAL have also been assessed in three small studies, which demonstrated no significant change in body fat with RAL over 48 weeks compared to N(t)RTI/PI based regimens [16], [19] or comparable increases in body fat to efavirenz [14]. More recently the larger PROGRESS study 96 week results demonstrated lopinavir/ritonavir (r/LPV) plus RAL increased peripheral fat, but not trunk fat compared to r/LPV plus tenofovir/emtricitabine [20].

The Metabolic Syndrome is a condition characterised by the clustering of alterations in glucose metabolism, lipid metabolism, fat accumulation and blood pressure. Several studies have reported a high prevalence of the Metabolic Syndrome in HIV populations [21]–[24], which may be due to cART associated lipid and adipose tissue disturbances. In one study, investigators established that after initiation of cART the incidence of Metabolic Syndrome was associated with significantly poorer CVD outcomes [24]. The Metabolic Syndrome has been identified as a significant risk factor for CVD by the U.S. National Cholesterol Education Program Adult Treatment Panel III (ATPIII) report [25], [26]. To date the effects of RAL on the Metabolic Syndrome compared to standard N(t)RTI/PI regimens has not been investigated.

CVD accounts for 10% of deaths in patients with HIV infection [27], which may be driven by HIV infection itself [28], lifestyle factors [29], [30] as well as cART [31]–[35]. There is a paucity of data evaluating the effect of RAL on adverse cardiac outcomes. One study conducted in healthy volunteers were given a supratherapeutic dose of RAL and demonstrated no prolongation of the QT interval, i.e. ventricular repolarization [36]. In addition, the PROGRESS study reported RAL did not significantly change the CVD risk in patients over 48 weeks [15].

The Second-Line study provided a unique opportunity to examine the lipodystrophy syndrome and CVD risk, using RAL + r/LPV as an alternate N(t)RTI-sparing treatment option for participants failing first-line NNRTI +2N(t)RTI. We hypothesised that an N(t)RTI-sparing cART regimen containing RAL would result in greater restoration of limb fat than combinations containing N(t)RTI.

## Materials and Methods

### Design

The Second-line study is a 96 week, multinational trial of participants failing first-line therapy, randomised 1∶1 and stratified by clinical site and screening plasma viral load (≤100 000 copies per mL or >100 000 copies per mL) to the World Health Organization recommended second-line treatment (r/LPV+2–3N(t)RTI) or r/LPV (400/100 mg bd or 800/200 mg qd)+ RAL (400 mg bd). The randomisation sequence was computer generated with blocked randomisation (block size of four) and triggered by the investigator entering all participant consent, screening, and eligibility data. Allocation was concealed until interventions were assigned, after which participants and investigators were not masked to treatment. Eligible participants were HIV-1 positive adults (aged ≥16 years) who had received first-line cART comprised of an NNRTI+2N(t)RTIs for ≥24 weeks with no change within 12 weeks prior to screening; evidence of virological failure defined by 2 consecutive (≥7 days apart) plasma HIV RNA viral load >500 copies per mL; no previous exposure to PIs and/or Integrase Strand Transfer Inhibitors. The study was approved by each site's Ethics Committee and was registered at Clinicaltrials.gov (NCT00931463). The cohort median (IQR) age was 38.5 (32.4–44.4) years, 55% male, 42% Asian and 36% African, 73% heterosexual transmission, with an estimated duration of HIV infection of 6.0 (3.6–8.7) years. The primary results of the Second Line study have been described [37].

The protocol and analysis plan for this trial and supporting CONSORT checklist are available as supporting information; see CONSORT checklist (Checklist S1), Bone and Body Comp Substudy protocol (Protocol S1), and SECONDLINE w48 bone and body comp analysis plan (Analysis Plan S1). Of the 37 sites that participated in the Second-Line study 8 sites from 5 countries (South Africa, India, Malaysia, Thailand, Argentina) participated in the body composition sub-study (clinicaltrials.gov identifier: NCT01513122) and analysed as a subgroup of the parent Second-Line study. These sites had access to a Dual energy X-ray absorptiometry (DXA) scanner and recruitment was open to all participants screened at these sites between July 2010 and July 2011. The sub-study was approved by each site's Ethics committee's and all participants gave written, informed consent. The specific ethic's committee's that gave approval for this sub-study are: **1.** YRG-CARE Institutional Review Board, Chennai, India 2. Medical Ethics Committee, University of Malaya Medical Centre, Kuala Lumpur, Malaysia 3. Kohn Kaen University Institutional Review Board, Kohn Kaen, Thailand 4. University of the Free State Ethics Committee, Bloemfontain, South Africa 5. Institutional Review Board, Faculty of Medicine, Chulalongkorn University, Bangkok, Thailand 6. Human Research Ethics Committee, Faculty of Health Sciences, University of Cape Town, Cape Town, South Africa 7. Human Research Ethics Committee (Medical), University of the Witwatersrand, Johannesburg, South Africa 8. Comite de Bioetica, CAEDI, Buenos Aires, Argentina. DXA scans were performed at baseline and week 48 on either Lunar (India n = 48, Malaysia n = 13, Argentina n = 8, Thailand n = 22) or Hologic (Thailand n = 26, South Africa n = 94) DXA scanners. Whole body composition was measured as per a standard protocol provided to all sites. We did not use phantoms for quality assurance and scans were not subjected to central interpretation, however scans were done on the same machine for each participant and all imaging centres had quality control measures in place.

The primary objective was to determine the difference in mean limb fat changes (absolute and percentage change) as measured by DXA scan between r/LPV+N(t)RTI and r/LPV+RAL arms from baseline to 48 weeks. The secondary objectives included comparisons between treatment arms for mean change in total body fat and trunk fat, distribution of limb fat percent change by treatment arm, changes in lipid and glucose parameters, changes in 10 year cardiovascular risk using the Framingham Equation [38], changes in prevalence of the Metabolic Syndrome [39], and to explore the relationship between limb fat mass and baseline variables.

### Statistical Analysis

Analyses included all participants consented to the body composition sub-study, who underwent randomisation, received at least one dose of study medication and who completed both week 0 and 48 DEXA scans. Results were considered statistically significant at a two sided α = 0.05. A sample size of 100 per randomised treatment arm was required to achieve 80% power to detect a mean difference of 1 kg in limb fat.

At baseline there were imbalances between the two treatment arms for gender, BMI and smoking status. All analyses were adjusted for the imbalances in these covariates. Linear regression was used to compare adjusted means of differences (baseline to week 48) between randomised arms. Logistic regression was used to compare the adjusted proportion of participants with Metabolic Syndrome at week 48. Backward stepwise linear regression was used to determine risk factors for limb fat change at week 48. Any variables which were significant in univariate analyses with p<0.10 were then included in multivariate analyses. The baseline covariates considered were age, gender, ethnicity, body mass index (BMI), smoking, blood pressure; concomitant medication (anti-hypertensive medication, lipid-lowering therapy); HIV and antiretroviral therapy markers (randomised treatment arm, HIV duration, CDC category, CD4+ and CD8+ lymphocyte counts, duration of antiretroviral therapy (ART), use of ta-NRTI vs non-thymidine NRTI, duration of ta-NRTI use; plasma HIV RNA); body composition (total lean mass and limb fat); metabolic markers (total cholesterol, high density lipoprotein (HDL) cholesterol, low density lipoprotein (LDL) cholesterol, triglycerides, total chol:HDL ratio); and glycaemic markers (glucose, insulin, and homeostatis model assessment (HOMA) -calculated insulin sensitivity). Age, gender and ethnicity remained in the multivariate model regardless of the univariate results due to the confounding influence of these parameters on limb fat.

## Results

**Figure 1:**
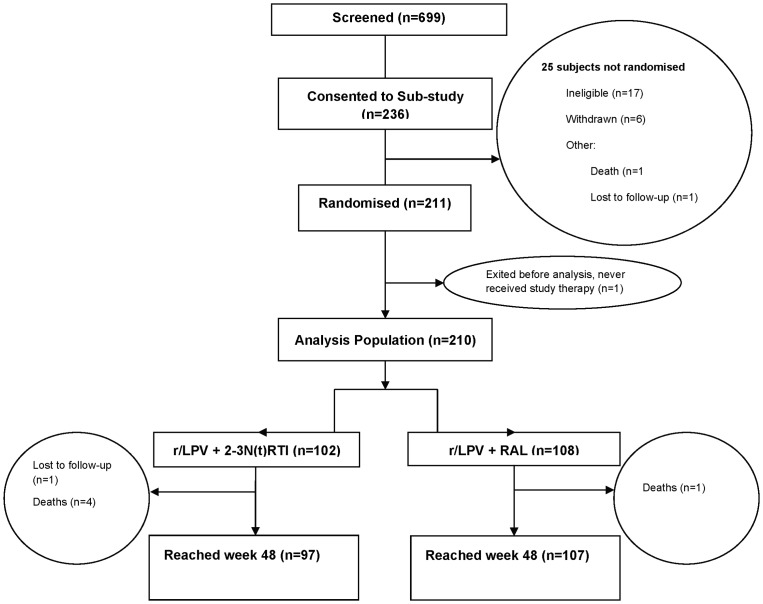
Patient disposition of SECONDLINE body composition sub-study.

Patient disposition is outlined in Figure 1. 699 participants were screened for the parent study, of whom 236 consented to the body composition sub-study. 211 participants were eligible and randomised into the sub-study and 210 made up the analysis population. 97 participants reached week 48 in the r/LPV+N(t)RTI arm and 107 in the r/LPV+RAL arm.

Baseline characteristics of the sub-study cohort are described in Table 1. The median age of the sub-study cohort was 38.8 years, 48% were male, 51% were Asian and 43% were African. At baseline the median total body fat was 29% (18–39%) and limb fat mass was 6.9 kg (3.9–11.1 kg). The median total cholesterol was 4.3 mmol/L (3.8–5.0 mmol/L), HDL cholesterol 1.1 mmol/L (0.9–1.4 mmol/L), and triglycerides were 1.3 mmol/L (0.9–2.1 mmol/L) at baseline. At baseline, 94% of the cohort were at low (<10%) risk of 10 year cardiovascular disease, 6% were at moderate risk and none had a high CVD risk. The prevalence of Metabolic Syndrome at baseline was 19% for the cohort.

**Table 1:** Baseline Characteristics.

	r/LPV +2–3N(t)RTI (n = 102)	r/LPV + RAL (n = 108)	Total (n = 210)
**Age, years**	38.6 (34.2–44.1)	38.9 (32.6–44.4)	38.8 (32.9–44.2)
**Sex, male**	55 (53.9)	45 (41.7)	100 (47.6)
**Ethnicity**			
Caucasian	4 (3.9)	3 (2.8)	7 (3.3)
Asian	53 (52.0)	55 (50.9)	108 (51.4)
Hispanic	1 (1.0)	2 (1.9)	3 (1.4)
African	44 (43.1)	47 (43.5)	91 (43.3)
Unknown	0	1 (0.9)	1 (0.5))
**Body Mass Index (BMI)**			
<18.5	18 (17.6)	13 (12.0)	31 (14.8)
18.5 to <20	48 (47.1)	59 (54.6)	107 (51.0)
20 to <30	26 (25.5)	24 (22.2)	50 (23.8)
30 to <35	7 (6.9)	8 (7.4)	15 (7.1)
≥35	3 (2.9)	4 (3.7)	7 (3.3)
**Hip/Waist ratio**	1.2 (1.1–1.2)	1.1 (1.1–1.2)	1.2 (1.1–1.2)
**Blood Pressure (mmHg)**	120/78 (109–126/70–81)	118/79 (109–129/70–83)	119/78 (109–128/70–82)
**HIV RNA log_10_ (copies/mL)**	4.3 (3.8–4.9)	4.1 (3.4–4.6)	4.1 (3.5–4.7)
**Total fat mass (kg)**	15.2 (8.3–22.1)	15.9 (10.0–23.8)	15.9 (8.8–22.7)
**Total fat mass (%)**	28 (15–35)	31 (18–40)	29 (18–39)
**Total lean mass (kg)**	40.3 (34.7–47.0)	40.8 (32.6–45.8)	40.6 (33.3–46.2)
**Limb fat mass (kg)**	6.3 (4.0–10.0)	7.4 (3.9–11.5)	6.9 (3.9–11.1)
**Limb fat mass (%)**	55 (20.3)	53 (19.6)	108 (20.0)
**Glucose (mmol/L)**	4.6 (4.3–5.1)	4.7 (4.3–5.1)	4.7 (4.3–5.1)
**Total Cholesterol (mmol/L)**	4.2 (3.6–5.0)	4.3 (3.8–4.9)	4.3 (3.8–5.0)
**HDL Cholesterol (mmol/L)**	1.1 (0.9–1.3)	1.1 (0.9–1.4)	1.1 (0.9–1.4)
**Total cholesterol:HDL ratio**	4.0 (3.2–5.0)	3.9 (2.9–4.7)	3.9 (3.0–4.8)
**LDL Cholesterol (mmol/L)**	2.5 (2.0–3.0)	2.6 (2.0–3.0)	2.5 (2.0–3.0)
**Triglycerides (mmol/L)**	1.3 (0.9–2.3)	1.3 (0.9–1.9)	1.3 (0.9–2.1)
**Insulin (mu/L)**	7.0 (4.6–11.9)	7.4 (4.5–14.0)	7.2 (4.5–13.5)
**HOMA**	1.4 (0.9–2.5)	1.6 (0.9–3.0)	1.5 (0.9–2.8)
**Framingham CVD 10 year risk**			
Low (<10%)	94 (93.1)	101 (95.3)	195 (94.2)
Moderate (10–20%)	7 (6.9)	5 (4.7)	12 (5.8)
High (>20%)	0	0	0
**Metabolic Syndrome n (%)**	23 (22.5)	17 (15.7)	40 (19.0)
**Smoking**			
Current	22 (21.6)	14 (13.0)	36 (17.1)
Recently (*within 12mth*)	1 (1.0)	2 (1.9)	3 (1.4)
Past	16 (15.7)	76 (70.4)	139 (66.2)
Never	63 (61.8)	52 (19.3)	87 (16.1)
**Alcohol consumption**			
0–2 drinks per day	97 (95.1)	104 (96.3)	201 (95.7)
≥2 drinks per day	5 (4.9)	4 (3.7)	9 (4.3)
**History of diabetes**	1 (1.0)	3 (2.8)	4 (1.9)
**Family history of diabetes**	20 (19.6)	25 (23.1)	45 (21.4)
**On TDF**	20 (19.6)	16 (14.8)	36 (17.1)
**On d4T**	50 (49.0)	51 (47.2)	101 (48.1)
**On AZT**	32 (31.4)	40 (37.0)	72 (34.3)
**Duration AZT (years)**	0.0 (0.0–2.1)	0.1 (0.0–2.3)	0.0 (0.0–2.2)
**Duration d4T (years)**	1.8 (0.0–3.4)	1.5 (0.0–3.8)	1.6 (0.0–3.6)

Data are median (IQR) or n (%); r/LPV: ritonavir boosted lopinavir; N(t)RTI: nucleoside reverse transcriptase inhibitor; RAL: raltegravir; TDF: tenofovir; d4T: stavudine; AZT: zidovudine.

**Figure 2:**
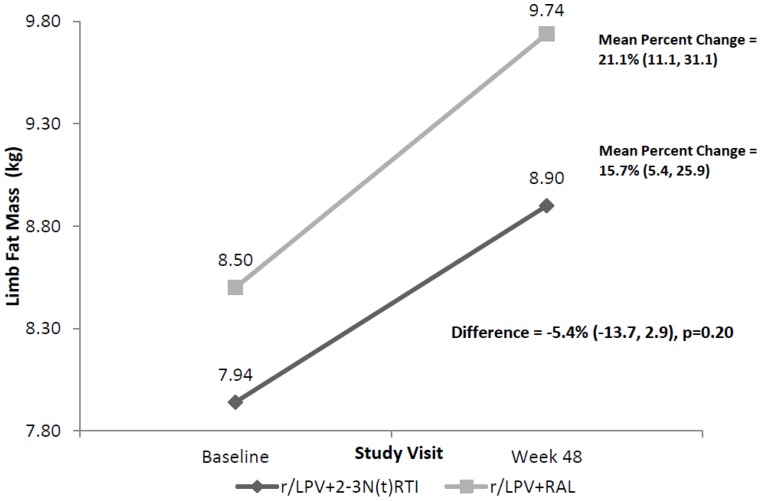
Mean change in limb fat mass from week

The primary objective results are outlined in Figure 2. Mean limb fat (95% CI) increased over the 48 weeks by 15.7% (5.4 to 25.9%) or 0.9 kg (0.2 to 1.5 kg) in the r/LPV+N(t)RTI arm compared with 21.1% (11.1 to 31.1%) or 1.3 kg (0.7 to 1.9) in the r/LPV+RAL arm. The mean difference was −5.4% (−13.7 to 2.9), p = 0.20.

The distribution of limb fat percent change by treatment arm is outlined in Table 2. 32% and 19% of participants experienced no increase in limb fat mass in the r/LPV+N(t)RTI and r/LPV+RAL arms, respectively. By contrast, 29% and 40% of participants in the r/LPV+N(t)RTI and r/LPV+RAL arms, respectively, had a >20% gain in limb fat mass.

**Table 2:** The distribution of percent limb fat gain by treatment arm.

Limb fat gain categories	r/LPV+2–3N(t)RTI (n = 94)	r/LPV+RAL (n = 107)	Total (n = 201)
≤0%	30 (31.9)	20 (18.7)	50 (24.9)
0.1–10%	22 (23.4)	31 (29.0)	53 (26.4)
10.1–20%	15 (16.0)	13 (12.2)	28 (13.9)
>20%	27 (28.7)	43 (40.2)	70 (34.8)

Data are expressed as n (%).

The total body and trunk fat mass increased in both treatment arms. Participants in the r/LPV+N(t)RTI arm experienced a mean (95% CI) increase in total body fat mass of 1.4 kg (0.2 to 2.7 kg) compared with changes in the r/LPV+RAL arm of 2.1 kg (0.9 to 3.3 kg), p = 0.20. Trunk fat mass increased by 0.6 kg (−0.1 to 1.2 kg) among recipients of r/LPV+N(t)RTI arm and by 0.8 kg (0.1 to 1.4 kg) among recipients of r/LPV+RAL arm, p = 0.40.

The mean changes over time in lipid and glucose parameters are summarised in Table 3. Triglycerides (r/LPV+N(t)RTI 0.6 mmol/L (0.3 to 0.9); r/LPV+RAL 0.8 (0.6 to 1.0) mean (95% CI)) and total cholesterol (r/LPV+N(t)RTI 0.4 (0.1 to 0.6); r/LPV+RAL 0.6 (0.4 to 0.9)) increased in both arms; however no significant between group differences were found (triglycerides −0.2 mmol/L (−0.6 to 0.2); p = 0.30; total cholesterol −0.3 mmol/L (−0.6 to 0.1); p = 0.13). HDL cholesterol increased in the r/LPV+N(t)RTI arm by 0.01 mmol/L (95% CI −0.1 to 0.1) but decreased in the r/LPV+RAL arm by 0.04 mmol/L (−0.1 to 0.0), with no significant differences between treatment arms (0.05 (−0.1 to 0.2); p = 0.32). Glucose decreased (r/LPV+N(t)RTI −0.04 mmol/L (−0.2 to 0.2); r/LPV+RAL −0.1 (−0.4 to 0.1)) but insulin (r/LPV+N(t)RTI 0.9mU/L (−0.5 to 2.3); r/LPV+RAL 0.9 (−0.6 to 2.5)) and HOMA (r/LPV+N(t)RTI 0.1 (−0.2 to 0.5); r/LPV+RAL 0.1 (−0.4 to 0.6) increased in both arms, however no significant between group differences were found (glucose 0.1 mmol/L (−0.2 to 0.4); p = 0.60; insulin −0.1 mU/L (−2.1 to 2.0); p = 0.95; HOMA 0.02 (−0.6 to 0.6); p = 0.93). The total:HDL cholesterol ratio increased to a statistically significant degree in the r/LPV+RAL arm (change over time 0.3 (0.1 to 0.6) r/LPV+N(t)RTI vs 0.7 (0.5 to 1.0) RAL arm, difference −0.4 (−0.8 to −0.04, p = 0.03).

**Table 3:** Changes from baseline to week

Metabolic Parameter	LPV/r+2–3N(t)RTI (n = 94)	LPV/r+RAL (n = 105)	P value
**Total cholesterol (mmol/L)**	0.3 (−0.2, 0.8)	0.5 (−0.2, 1.4)	0.27
**HDL cholesterol (mmol/L)**	0.0 (−0.2, 0.2)	0.0 (−0.2, 0.2)	0.52
**LDL cholesterol (mmol/L)**	0.1 (−0.2, 0.6)	0.3 (−0.2, 1.0)	0.17
**Triglycerides (mmol/L)**	0.3 (0.0, 1.1)	0.5 (0.1, 1.3)	0.12
**Total/HDL cholesterol ratio**	0.2 (−0.4, 0.8)	0.6 (−0.2, 1.3)	0.0209
**Glucose (mmol/L)**	−0.1 (−0.4, 0.4)	−0.1 (−0.4, 0.3)	0.97
**Insulin (mU/L)**	0.6 (−1.6, 4.0)	1.1 (−2.7, 4.8)	0.79
**HOMA**	0.1 (−0.5, 0.8)	0.2 (−0.6, 1.0)	0.65

Data are expressed as median (IQR).

The Metabolic Syndrome was assessed at baseline and week 48 in all sub-study participants. The proportion of participants with this syndrome at baseline was 23% in the r/LPV+N(t)RTI arm and 16% in the r/LPV+RAL arm. Throughout the study there were 8 new cases of Metabolic Syndrome in the r/LPV+N(t)RTI arm and 14 new cases in the r/LPV+RAL arm. There was no statistically significant difference between the newly acquired Metabolic Syndrome cases in each arm; OR (95% CI) 1.6 (0.6 to 4.0), p = 0.36.

The distribution of 10 year CVD risk categories for the sub-study cohort are summarised in Table 4. The majority (90–95%) of the cohort were at a low risk of experiencing a CVD event within 10 years at both baseline and week 48. Four participants in the r/LPV+N(t)RTI arm developed moderate risk of heart disease by week 48, while 5 participants in the r/LPV+RAL arm developed moderate risk and one participant developed high risk of heart disease by week 48. The mean change (95% CI) over 48 weeks in 10 year Framingham cardiovascular risk was −0.27% (−1.1 to 0.5) in the r/LPV+N(t)RTI arm and 0.26% (−0.5 to 1.1) in the r/LPV+RAL arm, which was not statistically significantly different between treatment arms, mean difference −0.52% (−1.2 to 0.1), p = 0.12.

**Table 4:** 10(Framingham Equation) categories by treatment arm at baseline and 48 weeks.

Treatment arm and Visit	Coronary Heart Disease Risk		
	Low (<10%)	Moderate (10–20%)	High (>20%)
**Baseline**			
r/LPV +2–3N(t)RTI	94 (93.1)	7 (6.9)	0
r/LPV + RAL	101 (95.3)	5 (4.7)	0
**Week 48**			
r/LPV +2–3N(t)RTI	86 (90.6)	9 (9.5)	0
r/LPV + RAL	99 (92.5)	7 (6.5)	1 (0.9)
**New Incidence at week 48**			
r/LPV +2–3N(t)RTI	0	4 (4.2)	0
r/LPV + RAL	0	5 (4.7)	1 (0.9)

Data are expressed as n (%).

The baseline covariates that were included in the multivariate regression are summarised in Table 5. The significant independent baseline predictors of gain in limb fat over the 48 week study were higher plasma HIV RNA (β 0.51, p = 0.0003) and higher insulin (β 0.06, p = 0.0012). Participants on lipid lowering therapy (β −1.68, p = 0.0286) at baseline were more likely to experience a reduction in limb fat.

**Table 5:** Baseline predictors of change in limb fat mass over 48

		Univariate		Multivariate	
		Limb Fat Change		Limb Fat Change	
Risk Factor (n = 201)	N	kg (95% CI)	P value	kg (95% CI)	P value
**Randomisation Arm**					
r/LPV +2–3N(t) RTI*	94				
r/LPV + RAL	107	0.46 (−0.06, 1.0)	0.0831	0.38 (−0.1, 0.9)	0.14
**Age** +	201	0.0057 (−0.03, 0.04)	0.74	0.02 (−0.02, 0.05)	0.37
**Sex** +					
Male*	94				
Female	107	0.32 (−0.2, 0.8)	0.22	0.39 (−0.2, 1.0)	0.22
**Race** +					
Caucasian*	6				
Asian	103	−0.19 (−1.2, 1.6)		−0.07 (−1.5, 1.4)	
Hispanic	3	−2.12 (−4.7, 0.4)		−1.73 (−4.2, 0.8)	
African Heritage	88	0.05 (−1.2, 1.7)	0.19	0.12 (−1.3, 1.6)	0.38
**Body mass index (kg/m^2^)** x	201	0.05 (−0.0,0.1)	0.0335	0.05 (−0.0,0.1)	0.13
**Smoking** x					
Currently*	34				
Recently	3	0.7 (−0.9, 3.5)		1.02 (−1.1, 3.2)	
Past	32	−0.05 (−1.0, 0.9)		−0.01 (−1.0, 0.9)	
Never	132	0.4 (−0.3, 1.1)	0.33	−0.01 (−0.8, 0.8)	0.81
**Glycaemic Markers**					
HOMA	197	−0.002 (−0.02, 0.2)	0.10	−0.09 (−0.3, 0.2)	0.49
Insulin (mU/L)	197	0.041 (0.006, 0.08)	0.0226	0.06 (0.0, 0.1)	0.0012
**Lipid lowering Therapy**					
No*	195				
Yes	6	−1.31 (−2.8, 0.2)	0.0891	−1.68 (−3.2, −0.2)	0.0286
**Log(HIV-RNA copies/mL)**	201	0.43 (0.2, 0.7)	0.0014	0.51 (0.2, 0.8)	0.0003

* reference group.

+ age, gender and ethnicity remained in the multivariate model regardless of the univariate results due to the confounding influence of these parameters on limb fat.

x body mass index and smoking were adjusted for due to baseline imbalance.

## Discussion

In this 210 participant sub-study of the Second-line trial we have demonstrated that in patients failing first line treatment comprising an NNRTI+2NtRTI, LPV/r plus RAL demonstrated similar improvements in lipoatrophy and did not significantly change CVD risk or the Metabolic Syndrome, compared to a LPV/r plus N(t)RTI regimen over 48 weeks. However, LPV/r plus RAL was associated with an increased total:HDL cholesterol ratio, suggesting an adverse affect on the lipid profile.

The majority of previous studies examining soft tissue changes associated with RAL treatment are limited by their small sample size. The STARTMRK [14] study reported an increase in limb fat of 18% and trunk fat 20% over 48 weeks with no further increases up to 96 weeks in 86 cART naive patients randomised to RAL + TDF/FTC fixed dose or efavirenz + TDF/FTC fixed dose, with no difference between treatment arms. These data are similar to the increase in fat mass reported in participants receiving RAL in our Second-line sub-study; 21% limb fat gain and 22% trunk fat. No change in body fat was reported in the SPIRAL-LIP sub-study when 74 virologically controlled HIV patients were randomised to either RAL or a PI [19] nor in the KITE study when 60 virologically controlled HIV patients were randomised to either RAL or N(t)RTI regimen [16]. More recently the PROGRESS study reported on 206 HIV ART naive patients randomised to LPV/r plus RAL or LPV/r plus tenofovir/emtricitabine over 96 weeks [20]. In this study participants randomised to the RAL arm significantly increased limb fat, but not trunk fat [20]. The reason for these differences may be explained by the different study populations. The STARTMRK, PROGRESS and Second-line trial participants were randomised with uncontrolled viral replication, the first two being cART naive and the last failing first line therapy, whereas SPIRAL-LIP and KITE patients were virologically controlled at randomisation. In addition, our study reported that high baseline viral load significantly predicted limb fat gain. This may infer that the increase in limb fat in the Second-line sub-study cohort was a ‘return to health’ after cART was switched to obtain virological control, especially since both the N(t)RTI arm and the N(t)RTI sparing arms experienced similar increases in limb fat mass. The significantly greater increase in peripheral fat reported in the PROGRESS, that was not found in our Second-line sub-study may be due to the different length of follow-up, 48 versus 96 weeks. Further sub-study analysis of the 96 week data is planned.

The Metabolic Syndrome was not significantly different over time between r/LPV+2–3N(t)RTI and r/LPV+RAL within the 48 weeks of this study. Metabolic Syndrome was numerically more prevalent in recipients of the r/LPV+RAL regimen compared to r/LPV+2–3N(t)RTI arm, however this was not a significant difference. Fat accumulation was seen in the limbs, trunk and over the total body with both treatment arms. Glucose, insulin and HOMA were similar between the groups, indicating that neither regimen adversely affected insulin sensitivity. Previous reports confirm this finding in that RAL has only mild affects (increase of 2 mg/dL) on glucose metabolism over 96 weeks [14]. Some lipid abnormalities have been previously reported with RAL, generally that RAL may cause an increase in total cholesterol and triglycerides but have no affect on total:HDL cholesterol ratio [15], [16], [37], [40]. When compared to efavirenz and PIs, RAL has been shown to have less effect on lipids [14], [17]. In comparison to the parent study total population [37], this sub-study did not demonstrate a significantly greater increase in total cholesterol, HDL, or LDL cholesterol with RAL; however it did find a significantly greater increase in the total:HDL cholesterol ratio compared with N(t)RTIs. This finding has not been reported previously and may be the component of the Metabolic Syndrome that caused the greater incidence in the RAL arm. This infers that the small (non-significant) increase in total cholesterol in the RAL arm was driven by a reduction in HDL cholesterol and an increase in LDL cholesterol.

The reasons for the different lipid results in this sub-study cohort compared to the parent study are unknown. There are some demographic differences between the parent study and the sub-study populations; one being the higher proportion of Asian and Africans in the sub-study (42% Asian, 36% African in the parent study vs 51% Asian, 43% African in the sub-study). Also, there was a higher proportion of women in the sub-study (52% vs 45%, sub-study vs parent study). Unfortunately, sub-analyses to investigate an association between ethnicity and gender are limited due to the small sample size of each sub-group and the small changes in lipid fractions. Analysis to assess the changes in lipid fractions over a longer period of time is needed to further investigate these cohort and lipid differences. Further sub-study analysis of the 96 week data is planned.

It is widely reported that there is a link between body fat mass, lipid abnormalities and cardiovascular disease in both non-HIV and HIV populations [25], [26], [41]. In this study limb fat mass increased after one year of RAL + r/LPV treatment, but the cholesterol ratio was worse. The finding that RAL has no major effect on CVD risk has previously been reported in the 48 week PROGRESS study [15]. This non-statistical CVD change in both studies may have been affected by too small a sample size to examine clinically significant changes in CVD risk. Larger and more detailed cardiovascular investigations would be needed, including assessment of cardiovascular biomarkers, to assess the long term affect of RAL on CVD.

Development of lipoatrophy is known to be associated with high HIV RNA [42]. In this sub-study it may also be true that patients with a higher HIV RNA at baseline have greater limb fat gain once their cART is switched and their viral load is controlled. The association reported in this sub-study between high baseline insulin and limb fat gain is interesting. The pathogenesis of insulin resistance may be through ectopic lipid accumulation in muscle and liver tissue, as well as by abnormalities in adipocytokine physiology in HIV patients with lipodystrophy [43]. In addition N(t)RTIs have been shown to cause insulin resistance, possibly though an indirect effect via the adipose tissue changes caused by N(t)RTIs [33]. Therefore, in this previously N(t)RTIs treated HIV population those with some degree of insulin resistance at baseline may gain limb fat to a greater degree because of the improvement in insulin sensitivity. Further and longer term analyses are needed to confirm this hypothesis, including thorough evaluation of insulin resistance at baseline. The finding that participants taking lipid lowering therapy at baseline were more likely to reduce limb fat over 48 weeks may suggest there is a direct affect of the concomitant medication on adipose tissue. Another reason may be that participants with lipid abnormalities (albeit controlled) are more likely to be previously exposed to ta-NRTIs and there is an intricate relationship between ta-NRTIs and inhibition of mitochondrial DNA polymerase γ with adipocytes.

This study was conducted primarily (94%) in an Asian (India, Thailand, Malaysia) and African (South Africa) population. To date there has been a paucity of data on body composition changes within the HIV populations of these countries. There have been two previous body composition studies reported in India, both using bioelectrical impedance analysers [44], [45]. These studies reported an increase in total body fat of 1.5 to 1.8 kg after 6 months of initiating first-line cART. These figures are similar to the results in our population which reported an increase in total body fat of approximately 2 kg over 48 weeks. There have also been two body composition studies reported in Thailand, using DXA [46], [47]. In both studies it was reported that limb fat increased by only 0.4 to 0.6 kg over 48 weeks after switching ART because of virological failure. One study in South Africa conducted on 83 ART naive HIV women investigated soft tissue changes using a DXA scanner and reported a total fat mass of 26 kg and trunk fat mass of 10 kg, which compares similarly with our population results of 17 kg total fat mass and 9 kg trunk fat mass. Therefore, the body composition data presented in this Second-line sub-study helps strengthen the evidence base for populations in which HIV infection is endemic and long-term co-morbidities are becoming a larger part of patient management as more cART are rolled out within the health systems.

In conclusion, this study suggests a switch to an N(t)RTI-sparing cART regimen consisting of r/LPV plus RAL has a similar affect on limb fat and cardiovascular disease risk compared with r/LPV plus N(t)RTIs, but may worsen the lipid profile.

## Supporting Information

Checklist S1 **CONSORT checklist.**
(DOC)

Protocol S1 **Bone and Body Comp Substudy Protocol.**
(PDF)

Analysis Plan S1 **SECONDLINE w48 bone and body comp analysis plan.**
(DOC)

## References

[1] Carr A, Samaras K, Burton S, Law M, Freund J, et al (1998) A syndrome of peripheral lipodystrophy, hyperlipidaemia and insulin resistance in patients receiving HIV protease inhibitors. *AIDS* 12: F51. 9619798 10.1097/00002030-199807000-00003

[2] Carr A, Miller J, Law M, Cooper DA (2000) A syndrome of lipoatrophy, lactic acidaemia and liver dysfunction associated with HIV nucleoside analogue therapy: contribution to protease inhibitor-related lipodystrophy syndrome. *AIDS* 14: F25. 10716495 10.1097/00002030-200002180-00001

[3] Bernasconi E, Boubaker K, Junghans C, Flepp M, Furrer HJ, et al (2002) Abnormalities of body fat distribution in HIV-infected persons treated with antiretroviral drugs: The Swiss HIV Cohort Study. *JAIDS* 31: 50. 12352150 10.1097/00126334-200209010-00007

[4] Lichtenstein KA, Ward DJ, Moorman AC, Delaney KM, Young B, et al (2001) Clinical assessment of HIV-associated lipodystrophy in an ambulatory population. *AIDS* 15: 1389–1398. 11504960 10.1097/00002030-200107270-00008

[5] Van der Valk M, Gisolf E, Reiss P, Wit F, Japour A, et al (2001) Increased risk of lipodystrophy when nucleoside analogue reverse transcriptase inhibitors are included with protease inhibitors in the treatment of HIV-1 infection. *AIDS* 15: 847. 11399957 10.1097/00002030-200105040-00005

[6] Martínez E, Mocroft A, García-Viejo MA, Pérez-Cuevas JB, Blanco JL, et al (2001) Risk of lipodystrophy in HIV-1-infected patients treated with protease inhibitors: a prospective cohort study. *Lancet* 357: 592–598. 11558485 10.1016/S0140-6736(00)04056-3

[7] Mallon PWG, Miller J, Cooper DA, Carr A (2003) Prospective evaluation of the effects of antiretroviral therapy on body composition in HIV-1-infected men starting therapy. *AIDS* 17: 971–979. 12700446 10.1097/00002030-200305020-00005

[8] Dubé MP, Parker RA, Tebas P, Grinspoon SK, Zackin RA, et al (2005) Glucose metabolism, lipid, and body fat changes in antiretroviral-naive subjects randomized to nelfinavir or efavirenz plus dual nucleosides. *AIDS* 19: 1807–1818. 16227788 10.1097/01.aids.0000183629.20041.bb

[9] Palella FJ, Cole SR, Chmiel JS, Riddler SA, Visscher B, et al (2004) Anthropometrics and examiner-reported body habitus abnormalities in the multicenter AIDS cohort study. *CID* 38: 903–907. 10.1086/381684 14999638

[10] Wand H, Law M, Emery S, Cooper D, Carr A (2005) Increase in limb fat after nucleoside analogue cessation is not associated with decreased visceral fat and has different risk factors. *Antivir Ther* 10: L5.

[11] Mallal SA, John M, Moore CB, James IR, McKinnon EJ (2000) Contribution of nucleoside analogue reverse transcriptase inhibitors to subcutaneous fat wasting in patients with HIV infection. *AIDS* 14: 1309–1316. 10930144 10.1097/00002030-200007070-00002

[12] Miller KD, Jones E, Yanovski JA, Shankar R, Feuerstein I, et al (1998) Visceral abdominal-fat accumulation associated with use of indinavir. *Lancet* 351: 871–875. 9525365 10.1016/S0140-6736(97)11518-5

[13] Bacchetti P, Gripshover B, Grunfeld C, Heymsfield S, McCreath H, et al (2005) Fat distribution in men with HIV infection. *JAIDS* 40: 121. 16186728 10.1097/01.qai.0000182230.47819.aa PMC3166344

[14] Lennox JL, DeJesus E, Berger DS, Lazzarin A, Pollard RB, et al (2010) Raltegravir versus efavirenz regimens in treatment-naive HIV-1-infected patients: 96-week efficacy, durability, subgroup, safety, and metabolic analyses. *JAIDS* 55: 39–48. 20404738 10.1097/QAI.0b013e3181da1287 PMC6065510

[15] Reynes J, Lawal A, Pulido F, Soto-Malave R, Gathe J, et al (2011) Examination of noninferiority, safety, and tolerability of lopinavir/ritonavir and raltegravir compared with lopinavir/ritonavir and tenofovir/emtricitabine in antiretroviral-naive subjects: the progress study, 48-week results. *HIV Clin Trials* 12: 255–267. 22180523 10.1310/hct1205-255

[16] Ofotokun I, Sheth AN, Sanford SE, Easley KA, Shenvi N, et al (2012) A switch in therapy to a reverse transcriptase inhibitor sparing combination of lopinavir/ritonavir and raltegravir in virologically suppressed HIV-infected patients: a pilot randomized trial to assess efficacy and safety profile: the KITE Study. *AIDS Res Hum Retro* 28: 1196–1206. 10.1089/aid.2011.0336 PMC3448110 22364141

[17] Martinez E, Larrousse M, Llibre JM, Gutierrez F, Saumoy M, et al (2010) Substitution of raltegravir for ritonavir-boosted protease inhibitors in HIV-infected patients: the SPIRAL study. *AIDS* 24: 1697. 20467288 10.1097/QAD.0b013e32833a608a

[18] Minami R, Yamamoto M, Takahama S, Ando H, Miyamura T, et al (2011) Comparison of the influence of four classes of HIV antiretrovirals on adipogenic differentiation: the minimal effect of raltegravir and atazanavir. *J Infect and Chemo* 17: 183–188. 10.1007/s10156-010-0101-5 20706762

[19] Curran A, Martinez E, Saumoy M, del Rio L, Crespo M, et al (2012) Body composition changes after switching from protease inhibitors to raltegravir: SPIRAL-LIP substudy. *AIDS* 26: 475. 22112606 10.1097/QAD.0b013e32834f3507

[20] Reynes J, Trinh R, Pulido F, Soto-Malave R, Gathe J, et al (2013) Lopinavir/Ritonavir Combined with Raltegravir or Tenofovir/Emtricitabine in Antiretroviral-Naive Subjects: 96-Week Results of the PROGRESS Study. *AIDS Res Hum Retro* 29: 256–265. 10.1089/aid.2011.0275 22730929

[21] Bruno R, Gazzaruso C, Sacchi P, Zocchetti C, Giordanetti S, et al (2002) High prevalence of metabolic syndrome among HIV-infected patients: link with the cardiovascular risk. *JAIDS* 31: 363–365. 12439215 10.1097/00126334-200211010-00015

[22] Gazzaruso C, Sacchi P, Garzaniti A, Fratino P, Bruno R, et al (2002) Prevalence of metabolic syndrome among HIV patients. *Diabet Care* 25: 1253–1254. 10.2337/diacare.25.7.1253 12087038

[23] Bonfanti P, Giannattasio C, Ricci E, Facchetti R, Rosella E, et al (2007) HIV and metabolic syndrome: a comparison with the general population. *JAIDS* 45: 426–431. 17514013 10.1097/QAI.0b013e318074ef83

[24] Wand H, Calmy A, Carey DL, Samaras K, Carr A, et al (2007) Metabolic syndrome, cardiovascular disease and type 2 diabetes mellitus after initiation of antiretroviral therapy in HIV infection. *AIDS* 21: 2445. 18025881 10.1097/QAD.0b013e3282efad32

[25] Grundy SM, Brewer HB Jr, Cleeman JI, Smith SC Jr, Lenfant C (2004) Definition of metabolic syndrome report of the National Heart, Lung, and Blood Institute/American Heart Association Conference on scientific issues related to definition. *Circulation* 109: 433–438. 14744958 10.1161/01.CIR.0000111245.75752.C6

[26] Pearson TA, Blair SN, Daniels SR, Eckel RH, Fair JM, et al (2002) AHA guidelines for primary prevention of cardiovascular disease and stroke: 2002 update consensus panel guide to comprehensive risk reduction for adult patients without coronary or other atherosclerotic vascular diseases. *Circulation* 106: 388–391. 12119259 10.1161/01.cir.0000020190.45892.75

[27] Department of Health and Human Services NIH (2012) AIDSinfo website. Guidelines for the use of antiretroviral agents in HIV-1 infected adults and adolescents. Available: http://aidsinfo.nih.gov/guidelines. Accessed 2013 Jun 8.

[28] Grunfeld C, Delaney JAC, Wanke C, Currier JS, Scherzer R, et al (2009) Pre-clinical atherosclerosis due to HIV infection: carotid intima-medial thickness measurements from the FRAM Study. *AIDS* 23: 1841. 19455012 10.1097/QAD.0b013e32832d3b85 PMC3156613

[29] Worm S, Friis-Moller N, Sabin C, Sjol A, Lundgren J, et al (2010) Factors associated with specific causes of death amongst HIV-positive individuals in the D: A: D study The Data Collection on Adverse Events of Anti-HIV drugs (D: A: D) Study Group. *AIDS* 24: 1537–1548. 20453631 10.1097/QAD.0b013e32833a0918

[30] Mamary EM, Bahrs D, Martinez S (2002) Cigarette smoking and the desire to quit among individuals living with HIV. *AIDS Pat Care and STDs* 16: 39–42. 10.1089/108729102753429389 11839217

[31] Stein JH, Currier JS (2008) Risk of myocardial infarction and nucleoside analogues. *Lancet* 371: 1391–1392. 18387666 10.1016/S0140-6736(08)60491-2

[32] Aboud M, Elgalib A, Pomeroy L, Panayiotakopoulos G, Skopelitis E, et al (2010) Cardiovascular risk evaluation and antiretroviral therapy effects in an HIV cohort: implications for clinical management: the CREATE 1 study. *Inter J Clin Prac* 64: 1252–1259. 10.1111/j.1742-1241.2010.02424.x PMC2913108 20653801

[33] Grinspoon S, Carr A (2005) Cardiovascular risk and body-fat abnormalities in HIV-infected adults. *NEJM* 352: 48–62. 15635112 10.1056/NEJMra041811

[34] Martin A, Bloch M, Amin J, Baker D, Cooper DA, et al (2009) Simplification of antiretroviral therapy with tenofovir-emtricitabine or abacavir-Lamivudine: a randomized, 96-week trial. *CID* 49: 1591. 10.1086/644769 19842973

[35] Friis-Moller N, Sabin C, Weber R, d'Arminio Monforte A, El-Sadr W, et al (2003) Combination antiretroviral therapy and the risk of myocardial infarction. *NEJM* 349: 1993–2003. 14627784 10.1056/NEJMoa030218

[36] Iwamoto M, Kost J, Mistry G, Wenning L, Breidinger S, et al (2008) Raltegravir thorough QT/QTc study: a single supratherapeutic dose of raltegravir does not prolong the QTcF interval. *J Clin Pharmacol* 48: 726–733. 18441333 10.1177/0091270008318007

[37] Boyd M (2013) SECOND-LINE: Ritonavir-Boosted-Lopinavir with 2–3N(t)RTI or Raltegravir in HIV-Positive Subjects Virologically Failing First-Line NNRTI/2N(t)RTI antiretroviral therapy. *Lancet* 381: 2091–99.23769235

[38] Wilson PWF, D'Agostino RB, Levy D, Belanger AM, Silbershatz H, et al (1998) Prediction of coronary heart disease using risk factor categories. *Circulation* 97: 1837–1847. 9603539 10.1161/01.cir.97.18.1837

[39] Alberti K, Zimmet P, Shaw J (2006) Metabolic syndrome–a new world-wide definition. A Consensus Statement from the International Diabetes Federation. *Diabet Med* 23: 469–480. 16681555 10.1111/j.1464-5491.2006.01858.x

[40] Ramkumar K, Neamati N (2009) Raltegravir: The evidence of its therapeutic value in HIV-1 infection. *Core Evid* 4: 131. 10.2147/ce.s6004 PMC2899791 20694070

[41] Kotler DP, Ionescu G, Johnson JA, Inada Y, He Q, et al (2003) Studies of Adipose Tissue Metabolism in Human Immunodeficiency Virus–Associated Lipodystrophy. *CID* 37: S47–S51. 10.1086/375891 12942374

[42] Lichtenstein KA, Delaney KM, Armon C, Ward DJ, Moorman AC, et al (2003) Incidence of and risk factors for lipoatrophy (abnormal fat loss) in ambulatory HIV-1-infected patients. *JAIDS* 32: 48–56. 12514413 10.1097/00126334-200301010-00007

[43] Hadigan C, Kamin D, Liebau J, Mazza S, Barrow S, et al (2006) Depot-specific regulation of glucose uptake and insulin sensitivity in HIV-lipodystrophy. *Am J Physiol-Endocrinol Metab* 290: E289–E298. 16131513 10.1152/ajpendo.00273.2005 PMC3197775

[44] Gupta V, Biswas A, Sharma S (2011) Metabolic and body composition changes after six months of highly active antiretroviral therapy in northern Indian patients. *Inter J STD & AIDS* 22: 46–49. 10.1258/ijsa.2010.010193 21364067

[45] Saghayam S, Kumarasamy N, Cecelia AJ, Solomon S, Mayer K, et al (2007) Weight and body shape changes in a treatment-naive population after 6 months of nevirapine-based generic highly active antiretroviral therapy in South India. *CID* 44: 295–300. 10.1086/510491 17173234

[46] Ananworanich J, Nuesch R, Côté HC, Kerr SJ, Hill A, et al (2008) Changes in metabolic toxicity after switching from stavudine/didanosine to tenofovir/lamivudine–a Staccato trial substudy. *J Antimicrob Chemo* 61: 1340–1343. 10.1093/jac/dkn097 18339636

[47] Boyd MA, Carr A, Ruxrungtham K, Srasuebkul P, Bien D, et al (2006) Changes in body composition and mitochondrial nucleic acid content in patients switched from failed nucleoside analogue therapy to ritonavir-boosted indinavir and efavirenz. *J Infect Dis* 194: 642–650. 16897663 10.1086/505709

