# Supplementary material for: HIV Lipodystrophy in Participants Randomised to Lopinavir/Ritonavir (LPV/r) +2–3 Nucleoside/Nucleotide Reverse Transcriptase Inhibitors (N(t)RTI) or LPV/r + Raltegravir as Second-Line Antiretroviral Therapy
Source: PLoS One. 2013 Oct 30;8(10):e77138. doi: 10.1371/journal.pone.0077138 (PMC3813715; doi:10.1371/journal.pone.0077138)
Supplement: Protocol S1 — Bone and Body Comp Substudy Protocol. (PDF) [file pone.0077138.s002.pdf]

NATIONAL CENTRE IN HIV
EPIDEMIOLOGY AND
CLINICAL RESEARCH

Clinical Protocol:

A randomised open-label study comparing the safety and efficacy of ritonavir boosted lopinavir and 2-3N(t)RTI backbone versus ritonavir boosted lopinavir and raltegravir in participants virologically failing first-line NNRTI/2N(t)RTI therapy: the SECOND-LINE study

Bone and Body Comp: A sub study of the SECOND-LINE study

Version 1.0

8th February 2010

Name and address of sponsor:

National Centre in HIV Epidemiology and Clinical Research (NCHECR)

University of New South Wales (UNSW)

Sydney, Australia

Telephone: +61 2 9385 0900

Facsimile: +61 2 9385 0910

Email: SECONDLINE@nchecr.unsw.edu.au

Principal investigator:

Professor David A Cooper

Professor of Medicine

National Centre for HIV Epidemiology and Clinical Research

University of New South Wales

Sydney, Australia, NSW, 2010.

1. SYNOPSIS 3

2. INTRODUCTION 5
2.1. Background 5
2.2. Body fat alterations 5
2.3. Bone alterations 6
2.4. Study rationale 6

3. RESEARCH HYPOTHESES 6

4. OBJECTIVES 6
4.1 Primary Objective 6
4.2 Secondary Objectives 7

5. STUDY POPULATION 8

6. METHODOLOGY 8
6.1 Clinical Demographics and Fracture Risk 8
6.2 DXA scan 8
6.2.1 Body Composition 8
6.2.2 Bone Mineral Density 8
6.3 Sample storage 9

7. STATISTICAL ANALYSIS 9

8. SAMPLE SIZE 10

9. FUNDING 10

10. REFERENCES 10

Appendix 1. Lunar DXA procedure for whole body, proximal femur and lumbar spine scans

Appendix 2. Hologic DXA procedure for whole body, proximal femur and lumbar spine scans

Appendix 3. Sample patient information statement and consent form.

1. SYNOPSIS

Title	Bone and Body Comp: A sub study of the SECOND-LINE study
Protocol Number	Version 1.0
Participating sites	This sub-study is open to sites participating in the SECOND-LINE study.
Patient Population	HIV-infected subjects enrolled into the SECOND-LINE study.
Treatment arms and duration	Arm 1. Lopinavir / ritonavir + 2-3N(t)RTI<br>Arm 2. Lopinavir /ritonavir + raltegravir<br>Subjects will be followed for 96 weeks.
Research Hypotheses	Body Fat:<br>HIV-infected subjects randomised into the experimental arm (LPV/r + RAL) will demonstrate greater increases in limb fat over 48 weeks than those randomised into the control arm (LPV/r + 2-3N(t)RTIs).<br>Bone:<br>HIV-infected subjects randomised into the experimental arm (LPV/r + RAL) will demonstrate smaller reductions in BMD at the proximal femur over 48 weeks than those randomised into the control arm (LPV/r + 2-3N(t)RTIs).
Primary Objectives	Body Fat:<br>To determine the difference in mean limb fat changes (absolute and percentage change) as measured by DXA scan between LPV/r + 2-3N(t)RTI and LPV/r + raltegravir based ART from baseline to 48 weeks.<br>Bone:<br>To determine the difference in BMD changes at the proximal femur (absolute and percentage change) as measured by DXA scan between LPV/r + 2-3N(t)RTI and LPV/r + raltegravir based ART from baseline to 48 weeks.
Secondary objectives	Body Fat:<br>To determine the mean change in regional and total body fat between LPV/r + 2-3N(t)RTI and LPV/r + raltegravir based ART as measured by DXA from baseline to weeks 48 and 96.<br>To determine the percentage of study subjects with >20% alterations in limb fat over 48 and 96 weeks between LPV/r + 2-3N(t)RTI and LPV/r + raltegravir based ART as measured by DXA .<br>Bone:<br>To determine and compare the mean percentage change in proximal femur and lumbar spine BMD between LPV/r + 2-3N(t)RTI and LPV/r + raltegravir based ART as measured by DXA from baseline to weeks 48 and 96.<br>To determine and compare the percentage of participants with low BMD (Z-score less than -2), osteopenia (T-score between -1.0 and -2.5) and osteoporosis (T-score less than -2.5) between LPV/r + 2-3N(t)RTI and LPV/r + raltegravir based ART from baseline to 96 weeks.<br>To determine and compare the mean percentage change from baseline in Z-score and T-score between LPV/r + 2-3N(t)RTI and LPV/r +

	raltegravir based ART as measured by DXA from baseline to weeks 48 and 96.<br><br>To estimate and compare changes in 10-year fracture risk using the FRAX equation between LPV/r + 2-3N(t)RTI and LPV/r + raltegravir based ART from baseline to 96 weeks.<br><br>To determine the added predictive value for fracture risk that is provided by BMD assessment using the FRAX® risk assessment tool.<br><br>To relate changes in BMD to changes in bone metabolism biomarkers (osteocalcin, CTX, OPG, RANK/L, vitamin D and parathyroid hormone), body composition (total and regional body fat) and clinical demographics.
Trial plan	Collection of demographic and bone specific clinical information, sample collection and whole body DXA and DXA of proximal femur and lumbar spine will be performed at baseline, week 48 and week 96.
Statistical analyses	Body fat:<br>The primary comparison will be percentage change in limb fat from baseline to w48. Secondary analyses will include between-group differences in change from baseline (to weeks 48 and 96), change between weeks 48 and 96 in total fat, trunk fat and lean mass, proportion of patients experiencing >20% reduction in limb fat (with and without corresponding changes in trunk fat) and proportion of patients meeting the definition of lipodystrophy according to the Lipodystrophy Case Definition Score.<br><br>Bone:<br>The primary between-group comparison will be percentage change proximal femur BMD from baseline to w48. Secondary analyses will include between-group differences in change from baseline (to weeks 48 and 96) and change between weeks 48 and 96 in BMD, T-score and Z score at proximal femur and lumbar spine, percentage of patients with diagnosis of low BMD (by Z-score), osteopenia and osteoporosis (by T-score) at proximal femur and lumbar spine and incident fractures.<br><br>Changes in BMD and regional and total body fat will be correlated to determine if there are relationships between changes in body fat and BMD after correction for clinical demographics and risk factors for low BMD.<br><br>Additional analyses include estimation of 10-year risk for osteoporotic fracture using FRAX® risk calculator both with and without BMD data to estimate the change in fracture risk before and after inclusion of BMD. A series of reclassification measures will then be utilised to quantify the added predictive value for fracture risk that is provided by BMD assessment.<br><br>Analysis will be primarily by intent-to-treat. A per-protocol analysis will also be performed.<br><br>Endpoints will be analysed using ANOVA methods or non-parametric equivalents with no adjustments made for multiple comparisons.
Site requirements	DXA scan (Hologic / LEXOSS / lunar or equivalent) with software capabilities to perform both whole body and regional DXA scans.

SECOND LINE STUDY

BONE AND BODY COMPOSITION SUBSTUDY

2. INTRODUCTION

2.1. Background

The advances in the treatment of HIV-infected individuals, particularly since the introduction of highly active anti-retroviral therapy (HAART), have significantly decreased the morbidity and mortality of this infection. However, excess mortality and morbidity from non-AIDS illnesses in HIV-infected patients is of ongoing concern, with conditions such as cardiovascular disease, bone disease and abnormalities of fat and lipid metabolism prevalent in HIV-infected patients. Both HIV infection itself and long term exposure to antiretrovirals (ART) have been implicated in the pathogenesis of these common conditions; body fat abnormalities (1-4) and bone alterations (5-8) can affect as many as 50% of individuals receiving ART.

2.2. Body fat alterations

Abnormalities of fat and lipid metabolism have long been linked to use of ART. Loss of subcutaneous adipose tissue (lipoatrophy) is associated with exposure to thymidine analogue nucleoside reverse transcriptase inhibitors (tNRTI) (1-4, 9-12) whilst changes in circulating lipoproteins have been demonstrated with use of all three major classes of ART (protease inhibitors [PI], NRTI and non-nucleoside reverse transcriptase inhibitors [NNRTI]) although the pattern of changes differ between the three classes (9, 13-17).

Although exposure to PI was initially thought to drive development of lipoatrophy, subsequent data suggested a more important role for tNRTI its pathogenesis and recent data suggest little potential for the development of lipoatrophy when PI are used without NRTI in antiretroviral naïve patients (Moyle, Castle Study, EACS 2009, LBPS11/6). Similarly, use of raltegravir in antiretroviral naïve patients has not been associated with significant changes in body composition (DeJesus, ICAAC 2009 H-1571).

In patients who have been exposed to NRTI, switching from tNRTI to non-thymidine NRTI improves limb fat (18-21) albeit slowly and with incomplete restoration for many patients. Less data is available on the effect of NRTI-sparing regimens on limb fat in patients previously exposed to NRTI (21-23) with most examining the combination of a PI + NNRTI, use of which is associated with a worrying trend toward increased virological failures (24-25).

2.3. Bone alterations

Low bone mineral density (BMD) and osteoporosis are prevalent in HIV-infected patients with more osteoporosis-related fractures experienced in HIV-infected patients than the general population. Although cross sectional studies suggest this increased prevalence to be associated both with HIV infection itself in addition to a greater prevalence of traditional risk factors for low BMD in HIV-infected patients (such as smoking, low BMI and steroid use), studies in ART naïve patients initiating ART have consistently demonstrated loss of BMD over the first 48 weeks of exposure to ART, with greater loss experienced by those initiating tNRTI and PI. Little is known on the effects of exposure to integrase inhibitors and BMD.

It is unclear if this initial loss of BMD is related to exposure to ART, immune restoration or arises as a consequence of altered inflammatory responses resulting from control of HIV viraemia. If the latter is the case, then additional loss of BMD may occur in the setting of virological failure and use of second line ART.

2.4. Study rationale

The SECOND-LINE study provides an opportunity to examine if NRTI-sparing ART regimens containing integrase inhibitors (experimental arm of SECOND-LINE [LPV/r + RAL) result in greater restoration of limb fat than combinations containing NRTI (control arm of SECOND-LINE [LPV/r + 2-3N(t)RTI]) in HIV-infected patients previously exposed to NRTI.

This study also provides an opportunity to examine if additional bone loss occurs with second line treatment of HIV infection and whether NRTI-sparing ART used in second line therapy results in greater or less bone loss than use of PI/NRTI combinations (control arm of SECOND-LINE [LPV/r + 2-3N(t)RTI]).

3. RESEARCH HYPOTHESES

Body Fat:

HIV-infected subjects randomised into the experimental arm (LPV/r + RAL) will demonstrate greater increases in limb fat over 48 weeks than those randomised into the control arm (LPV/r + 2-3N(t)RTIs).

Bone:

HIV-infected subjects randomised into the experimental arm (LPV/r + RAL) will demonstrate smaller reductions in BMD at the proximal femur over 48 weeks than those randomised into the control arm (LPV/r + 2-3N(t)RTIs).

4. OBJECTIVES

4.1 Primary Objective

Body Fat:

- To determine the difference in mean limb fat changes (absolute and percentage change) as measured by DXA scan between LPV/r + 2-3N(t)RTI and LPV/r + raltegravir based ART from baseline to 48 weeks.

Bone:

- To determine the difference in BMD changes at the proximal femur (absolute and percentage change) as measured by DXA scan between LPV/r + 2-3N(t)RTI and LPV/r + raltegravir based ART from baseline to 48 weeks.

4.2 Secondary Objectives

Body Fat:

- To determine the mean change in regional and total body fat between LPV/r + 2-3N(t)RTI and LPV/r + raltegravir based ART as measured by DXA from baseline to weeks 48 and 96.
- To determine the percentage of study subjects with >20% alterations in limb fat over 48 and 96 weeks between LPV/r + 2-3N(t)RTI and LPV/r + raltegravir based ART as measured by DXA .

Bone:

- To determine and compare the mean percentage change in proximal femur and lumbar spine BMD between LPV/r + 2-3N(t)RTI and LPV/r + raltegravir based ART as measured by DXA from baseline to weeks 48 and 96.
- To determine and compare the percentage of participants with low BMD (Z-score less than -2), osteopenia (T-score between -1.0 and -2.5) and osteoporosis (T-score less than -2.5) between LPV/r + 2-3N(t)RTI and LPV/r + raltegravir based ART from baseline to 96 weeks.
- To determine and compare the mean percentage change from baseline in Z-score and T-score between LPV/r + 2-3N(t)RTI and LPV/r + raltegravir based ART as measured by DXA from baseline to weeks 48 and 96.
- To estimate and compare changes in 10-year fracture risk using the FRAX equation between LPV/r + 2-3N(t)RTI and LPV/r + raltegravir based ART from baseline to 96 weeks.
- To determine the added predictive value for fracture risk that is provided by BMD assessment using the FRAX® risk assessment tool.
- To relate changes in BMD to changes in bone metabolism biomarkers (osteocalcin, CTX, OPG, RANK/L, vitamin D and parathyroid hormone), body composition (total and regional body fat) and clinical demographics.

5. STUDY POPULATION

There will be approximately 52 investigative centres participating in the main Second Line protocol. All sites will be offered this sub-study but only those with the available infrastructure will participate. Pregnant or breast feeding mothers are excluded from entering the main protocol as well as this sub-study. If a woman becomes pregnant during the study, they will not participate in this sub-study.

6. METHODOLOGY

6.1 Clinical Demographics and Fracture Risk

In addition to demographics collected as part of the main study protocol, the following information will be collected at baseline; country of birth, ethnicity, socioeconomic status (employment status, maximum educational grade achieved, annual income, number of dependents) previous and incident fractures (including low-impact fractures) and risk factors for osteoporosis (smoking status, family history of hip fracture, hypogonadism, last menstrual period (to estimate onset of menopause / menarche), lifetime steroid exposure, alcohol intake and secondary causes of osteoporosis), recreational drug use (including anabolic steroids), medication exposure (including exposure to heparin, opiates and oestrogen replacement) and estimates of physical activity.

At weeks 48 and 96 the following additional information will be collected; socioeconomic status (employment status, maximum educational grade achieved, annual income, number of dependents), incident fractures (including low-impact fractures) change in risk factors for osteoporosis (smoking status, family history of hip fracture, hypogonadism, last menstrual period (to estimate onset of menopause / menarche), lifetime steroid exposure, alcohol intake and secondary causes of osteoporosis), recreational drug use (including anabolic steroids), medication exposure (including exposure to heparin, opiates and oestrogen replacement) and estimates of physical activity.

6.2 DXA scan

6.2.1 Body Composition

Whole body DXA will be performed at weeks 0, 48 and 96 using the protocol specified in appendix 1. Scans should be performed within 2 weeks of the study visit.

6.2.2 Bone Mineral Density

DXA scan for BMD of the proximal femur and lumbar spine will be performed at weeks 0, 48 and 96 using the protocol specified in appendix 2. Scans should be performed within 2 weeks of the study visit.

6.3 Sample storage

The list of biomarkers will be decided upon by the Bone and Body Comp sub group of the Protocol Steering Committee upon completion of the primary comparisons. Assays will be performed batched at the end of 96 weeks. These assays will be performed using stored samples already being collected as part of the main SECOND-LINE study protocol.

7. STATISTICAL ANALYSIS

Body fat:

The primary comparison will be percentage change in limb fat from baseline to w48.

Secondary analyses will include between-group differences in change from baseline (to weeks 48 and 96), change between weeks 48 and 96 in total fat, trunk fat and lean mass, the proportion of patients experiencing >20% reduction in limb fat (with and without corresponding changes in trunk fat) and the proportion of patients meeting the definition of lipodystrophy according to the Lipodystrophy Case Definition Score. Secondary analyses will control for age, gender and baseline BMI.

Bone:

The primary between-group comparison will be percentage change proximal femur BMD from baseline to w48. Secondary analyses will include between-group differences in change from baseline (to weeks 48 and 96) and change between weeks 48 and 96 in BMD, T-score and Z score at proximal femur and lumbar spine, percentage of patients with diagnosis of low BMD (by Z-score), osteopenia and osteoporosis (by T-score) at proximal femur and lumbar spine and incident fractures.

Changes in BMD and regional and total body fat will be correlated to determine if there are relationships between changes in body fat and BMD after correction for clinical demographics and risk factors for low BMD.

Additional analyses include estimation of 10-year risk for osteoporotic fracture using FRAX® risk calculator both with and without BMD data to estimate the change in fracture risk before and after inclusion of BMD. A series of reclassification measures will then be utilised to quantify the added predictive value for fracture risk that is provided by BMD assessment.

Analysis will be primarily by intent-to-treat (defined as all study subjects who undergo randomisation and who receive at least one dose of study medication) in which subjects will be compared regardless of the treatment received. A per-protocol analysis will also be performed (defined as all subjects from the intent-

to-treat population excluding those who changed randomly assigned antiretroviral therapy for any reason other than the HIV RNA exceeding 200 copies/ml). Endpoints will be analysed using ANOVA methods or non-parametric equivalents with no adjustments made for multiple comparisons.

8. SAMPLE SIZE

Target recruitment is 100 subjects per arm.

Body fat:

From data derived from the INITIO study, inclusion of 100 subjects per arm would provide 80% power to detect a mean difference between study arms of 1 kg (SD = 2.5 kg) in limb fat and 2.4% in limb fat percentage.

Bone:

Assuming a normal age related change in BMD of 1.8% over 96 weeks (0.9% per year is standard change in adults >40 years old) with a standard deviation of 6%, inclusion of 100 subjects per arm would provide 80% power to detect a between group difference in change in BMD over 48 and 96 weeks of 1.7%, a clinically relevant difference at the lower end of that observed in clinical trials (18, 20, 22).

9. FUNDING

The sponsor, NCHECR, will fund this study.

10. REFERENCES

1. Carr A, Samaras K, Burton S, et al. A syndrome of peripheral lipodystrophy, hyperlipidaemia and insulin resistance in patients receiving HIV protease inhibitors. Aids 1998; 12(7):F51-8.
2. Carr A, Miller J, Law M, Cooper DA. A syndrome of lipoatrophy, lactic acidaemia and liver dysfunction associated with HIV nucleoside analogue therapy: contribution to protease inhibitor-related lipodystrophy syndrome. Aids 2000;14(3):F25-32.
3. Bernasconi E, Boubaker K, Junghans C, et al. Abnormalities of body fat distribution in HIV-infected persons treated with antiretroviral drugs: The Swiss HIV Cohort Study. J Acquir Immune Defic Syndr 2002;31(1):50-5.
4. Lichtenstein KA, Ward DJ, Moorman AC, et al. Clinical assessment of HIV-associated lipodystrophy in an ambulatory population. Aids 2001;15(11):1389-98.
5. Tebas P, Powderly WG, Claxton SH, Marin D, Tantisiriwat W, Teitelbaum S, *et al*. Accelerated bone mineral loss in HIV-infected patients receiving potent antiretroviral therapy. *AIDS* 2000, 14: 3-67.
6. Moore AL, Vashisht A, Sabin C, Mocroft A, Madge S. Reduced bone mineral density in HIV-positive individuals. *AIDS* 2001, 15:1731-1733.
7. Nolan D, Upton R, McKinnon E**.** Longitudinal analysis of bone mineral density [DMO] in HIV-infected patients treated with HAART: change in subcutaneous fat; with an additional independent effect of indinavir therapy to increase DMO**.** *Antiviral Ther* 2000, 5(Suppl. 5):20.
8. Hoy J, Hudson J, Law M, Cooper DA. Osteopenia in a randomized multicenter study of protease inhibitor substitution in patients with the lipodystrophy syndrome and well - controlled HIV viremia.

Seventh Conference on Retroviruses and Opportunistic Infections. San Francisco, January-February 2000 [abstract 59].

9. Van der Valk M, Gisolf EH, Reiss P, et al. Increased risk of lipodystrophy when nucleoside analogue reverse transcriptase inhibitors are included with protease inhibitors in the treatment of HIV-1 infection. Aids 2001; 15(7):847-55.

10. Martinez E, Mocroft A, Garcia-Viejo MA, et al. Risk of lipodystrophy in HIV-1-infected patients treated with protease inhibitors: a prospective cohort study. Lancet 2001;357(9256):592-8.

11. Mallon PW, Miller J, Cooper DA, Carr A. Prospective evaluation of the effects of antiretroviral therapy on body composition in HIV-1-infected men starting therapy. Aids 2003;17(7):971-9.

12. Dube MP, Parker RA, Tebas P, et al. Glucose metabolism, lipid, and body fat changes in antiretroviral-naive subjects randomized to nelfinavir or efavirenz plus dual nucleosides. Aids 2005;19(16):1807-18.

13. Palella FJ, Jr., Cole SR, Chmiel JS, et al. Anthropometrics and examiner-reported body habitus abnormalities in the multicenter AIDS cohorte study. Clin Infec Dis 2004;38:903-907.

14. Wand H, Law M, Emery S, Cooper DA, Carr A. Increase in limb fat after nucleoside analogue cessation is not associated with decreased visceral fat and has different risk factors. 7th International Workshop on Adverse Drug Reactions and Lipodystrophy in HIV. Antivir Ther 2005; 10 Suppl 3:L1-L67.

15. Mallal SA, John M, Moore CB, James IR, McKinnon EJ. Contribution of nucleoside analogue reverse transcriptase inhibitors to subcutaneous fat wasting in patients with HIV infection. AIDS 2000; 14:1309-1316.

16. Miller KD, Jones E, Yanovski JA, Shankar R, Feuerstein I, Falloon J. Visceral abdominal-fat accumulation associated with tuse of indinavi. Lancet 1998; 351:871-875.

17. Bacchetti P, Gripshover B, Grunfeld C, et al. Fat distribution in men with HIV infection. J Acquir Immune Defic Sydnr 2005; 40:121-131.

18. Martin A, Smith DE, Carr A, et al. Reversibility of lipoatrophy in HIV-infected patients 2 years after switching from a thymidine analogue to abacavir: the MITOX Extension Study. AIDS. 2004;18:1029–1036.

19. McComsey GA, Ward DJ, Hessenthaler SM, et al. Improvement in lipoatrophy associated with highly active antiretroviral therapy in human immunodeficiency virus-infected patients switched from stavudine to abacavir or zidovudine: the results of the TARHEEL study. Clin Infect Dis. 2004;38:263–270.

20. Moyle G, Sabin C, Cartledge J, et al. A 48 week, randomized open-label comparative study of tenofovir DF vs. abacavir as substitutes for thymidine analogue in persons with lipoatrophy and sustained virologic suppression on HAART. Program and abstracts of the 12th Conference on Retroviruses and Opportunistic Infections; February 22-25, 2005; Boston, Massachusetts. Abstract 44LB.

21. Murphy R, Zhang J, Hafner R, et al. Switching to a thymidine analogue-sparing or a nucleoside-sparing regimen improves lipoatrophy: 24 week results of a prospective randomized clinical trial. AACTG 5110. Program and abstracts of the 12th Conference on Retroviruses and Opportunistic Infections; February 22-25, 2005; Boston, Massachusetts. Abstract 45LB.

22. Negredo E, Molto J, Burger D, et al. Lopinavir/ritonavir plus nevirapine as a nucleoside-sparing approach in antiretroviral-experienced patients (NEKA study). J Acquir Immune Defic Syndr. 2005;38:47–52.

23. Haubrich R, Riddler S, DiRienzo A, et al. Metabolic outcomes in a randomized trial of nucleoside, nonnucleoside and protease inhibitor-sparing regimens for initial HIV treatment. AIDS 2009; 23:1109-1118.

24. Fischl M, Bassett R, Collier A, et al. Randomized, controlled trial of lopinavir/ritonavir + efavirenz vs. efavirenz + 2 nucleoside reverse transcriptase inhibitors following a first suppressive 3- or 4- drug regimen in advanced HIV disease. Program and abstracts of the 12th Conference on Retroviruses and Opportunistic Infections; February 22-25, 2005; Boston, Massachusetts. Abstract 162.

25. Tebas P, Zhang J, Yarasheski K, et al. Switch to a protease inhibitor-containing/nucleoside reverse transcriptase inhibitor-sparing regimen increases appendicular fat and serum lipid levels without affecting glucose metabolism or bone mineral density. The results of a prospective randomized trial ACTG 5125s. Program and abstracts of the 12th Conference on Retroviruses and Opportunistic Infections; February 22-25, 2005; Boston, Massachusetts. Abstract 40.

26. Borderi M, Gibelline D, Vescini F, et al. Metabolic bone disease in HIV infection. AIDS 2009; 23:1297-1310.

27. Sambrook P, Cooper C. Osteoporosis. Lancet 2006; 367:2010–2018.

28. Anastos, K., et al., *The association of bone mineral density with HIV infection and antiretroviral treatment in women.* Antivir Ther, 2007. 12(7): p. 1049-58.
29. Gallant, J.E., et al., *Efficacy and safety of tenofovir DF vs stavudine in combination therapy in antiretroviral-naive patients: a 3-year randomized trial.* JAMA, 2004. 292(2): p. 191-

Appendix 1. Lunar DXA procedure for whole body, proximal femur and lumbar spine DXA scans .

Appendix 2. Hologic DXA procedure for whole body, proximal femur and lumbar spine DXA scans.

Appendix 3. Sample patient information statement and consent form.
