# Supplementary material for: HIV Lipodystrophy in Participants Randomised to Lopinavir/Ritonavir (LPV/r) +2–3 Nucleoside/Nucleotide Reverse Transcriptase Inhibitors (N(t)RTI) or LPV/r + Raltegravir as Second-Line Antiretroviral Therapy
Source: PLoS One. 2013 Oct 30;8(10):e77138. doi: 10.1371/journal.pone.0077138 (PMC3813715; doi:10.1371/journal.pone.0077138)
Supplement: Analysis Plan S1 — SECONDLINE w48 bone and body comp analysis plan. (DOC) [file pone.0077138.s003.doc]

**SECONDLINE Week 48 Bone and Body Composition Sub-study Analysis Plan**

**1. INTRODUCTION**

This analysis plan covers the Bone and Body Composition Sub-study of the SECOND-LINE study.

The analysis will be conducted when all participants who consented to the Bone and Body Composition Sub-study have been followed for at least 48 weeks, or have been permanently discontinued from the study.

# Normality of the distributions for each baseline variable will be checked by plotting histograms. If parameters are found to be not normally distributed then the equivalent non-parametric test will be used. Results will be considered statistically significant at a two sided α=0.05. Results should be interpreted with respect to clinical relevance as well as statistical significance. Baseline characteristics of this cohort will be described prior to analyses.

# 2. RESEARCH HYPOTHESES

Body Fat:

HIV-infected subjects randomised into the experimental arm (LPV/r + RAL) will demonstrate greater increases in limb fat over 48 weeks than those randomised into the control arm (LPV/r + 2-3N(t)RTIs).

Bone:

HIV-infected subjects randomised into the experimental arm (LPV/r + RAL) will demonstrate smaller reductions in BMD at the proximal femur over 48 weeks than those randomised into the control arm (LPV/r + 2-3N(t)RTIs).

**3. STUDY POPULATION**

Analyses will include all participants consented to the bone and body composition sub-study and who completed both a week 0 and 48 DEXA scan. Participants will be analysed in their randomised treatment group by intent-to-treat (defined as all study subjects who receive at least one dose of study medication). Primary analyses will only be adjusted for parameters reported to be imbalanced between the randomised groups at baseline.

**4. PRIMARY OBJECTIVES AND RELATED STATISTICAL ANALYSIS**

**4.1 BODY COMPOSITION**

***Objective 1. To determine the mean change in limb fat mass as measured by DXA scan between LPV/r + 2-3N(t)RTI and LPV/r + raltegravir based ART from baseline to 48 weeks.***

Total limb fat mass change will be defined as the following two parameters:

1. absolute from baseline to week 48
2. percent change in mass from baseline to week 48

Data will be described at baseline, at week 48 and then as the change from baseline to week 48. The distribution of these parameters will be summarised as mean, standard deviation, median, inter-quartile range, minimum and maximum. The distribution of these data will be presented graphically (either by histogram or box plot).

The null hypothesis is that there is no statistically significant difference between randomization arms in mean limb fat mass change (percentage and absolute) from baseline to week 48. Unpaired t-test will be used to compare means of differences (0 to 48) between randomization arms.

**4.2 BONE**

***Objective 1.*** ***To determine the mean change in BMD at the proximal femur as measured by DXA scan between LPV/r + 2-3N(t)RTI and LPV/r + raltegravir based ART from baseline to 48 weeks.***

BMD change will be defined as the following two parameters:

1. absolute from baseline to week 48
2. percent change from baseline to week 48

The distribution of these parameters will be summarised as mean, standard deviation, median, inter-quartile range, minimum and maximum. The distribution of these data will be presented graphically (either by histogram or box plot).

The null hypothesis is that there is no statistically significant difference between randomization arms in mean proximal femur BMD (absolute and percentage) from baseline to week 48. Unpaired t-test will be used to compare mean change between randomization arms.

**5. SECONDARY OBJECTIVES AND RELATED STATISTICAL ANALYSIS**

**5.1 BODY COMPOSITION**

***Objective 1. To determine the mean change in total body fat mass between LPV/r + 2-3N(t)RTI and LPV/r + raltegravir based ART as measured by DXA from baseline to week 48.***

Total body fat mass change will be defined as the following two parameters:

1. absolute from baseline to week 48
2. percent change in mass from baseline to week 48

Data will be described at baseline, at week 48 and then as the change from baseline to week 48. The distribution of these parameters will be summarised as mean, standard deviation, median, inter-quartile range, minimum and maximum. The distribution of these data will be presented graphically (either by histogram or box plot).

The null hypothesis is that there is no statistically significant difference between randomization arms in mean mass change (absolute and percentage) in total body fat from baseline to week 48. Unpaired t-test will be used to compare means between randomization arms.

***Objective 2. To describe the distribution of change in peripheral limb fat mass***

The distribution, number and proportion, of participants experiencing percent change in limb fat mass from baseline to week 48 according the following categories will be summarised by treatment arm and for the total cohort. These categories will only be descriptive, not statistically analysed as the numbers within each group will be small.

1. ≤0% peripheral fat gain
2. 0.1-10% peripheral fat gain
3. 10.1-20% peripheral fat gain
4. > 20% peripheral fat gain

***Objective 3. To determine the difference in mean trunk fat mass changes as measured by DXA scan between LPV/r + 2-3N(t)RTI and LPV/r + raltegravir based ART from baseline to 48 weeks.***

Central trunk fat mass change will be defined as the following two parameters:

1. absolute from baseline to week 48
2. percent change in mass from baseline to week 48

Data will be described at baseline, at week 48 and then as the change from baseline to week 48. The distribution of these parameters will be summarised as mean, standard deviation, median, inter-quartile range, minimum and maximum. The distribution of these data will be presented graphically (either by histogram or box plot).

The null hypothesis is that there is no statistically significant difference between randomization arms in mean change (absolute and percentage) in central trunk fat from baseline to week 48. Unpaired t-test will be used to compare means of differences (0 to 48) between randomization arms.

**5.2 BONE**

***Objective 1.*** ***To determine the mean change in BMD at the lumbar spine as measured by DXA scan between LPV/r + 2-3N(t)RTI and LPV/r + raltegravir based ART from baseline to 48 weeks.***

BMD change will be defined as the following two parameters:

1. absolute from baseline to week 48
2. percent change from baseline to week 48

The distribution of these parameters will be summarised as mean, standard deviation, median, inter-quartile range, minimum and maximum. The distribution of these data will be presented graphically (either by histogram or box plot).

The null hypothesis is that there is no statistically significant difference between randomization arms in mean lumbar spine BMD (absolute and percentage) from baseline to week 48. Unpaired t-test will be used to compare mean change between randomization arms.

***Objective 2. To determine and compare the percentage of participants with low BMD (Z-score less than -2), osteopenia (T-score between -1.0 and -2.5) and osteoporosis (T-score less than -2.5) between LPV/r + 2-3N(t)RTI and LPV/r + raltegravir based ART from baseline to 48 weeks.***

The distribution, number and proportion, of participants in the following categories will be summarised by treatment arm and for the total cohort. These categories will be assessed at both the hip and spine by McNemars test for paired proportions at week 0 and 48.

1. Low BMD = z score < -2
2. Osteopenia = t score between -1.0 and -2.5
3. Osteoporosis = t score <-2.5

***Objective 3. To determine and compare the mean absolute and percentage change from baseline in Z-score and T-score between LPV/r + 2-3N(t)RTI and LPV/r + raltegravir based ART as measured by DXA from baseline to weeks 48.***

The distribution of mean absolute and percentage change in z and t score at both the hip and spine from baseline to week 48 will be summarised as mean, standard deviation, median, inter-quartile range, minimum and maximum. The distribution of these data will be presented graphically (either by histogram or box plot).

The null hypothesis is that there is no statistically significant difference between randomization arms in mean absolute or percentage change in z and t score at both the hip and spine from baseline to week 48. Unpaired t-test will be used to compare mean percent change in z and t score between randomization arms.

**6.0 EXPLORATORY ANALYSES**

**6.1 BODY COMPOSITION**

***Objective 1. To estimate and compare changes in 10-year CVD risk using the Framingham equation between LPV/r + 2-3N(t)RTI and LPV/r + raltegravir based ART from baseline to 48 weeks.***

The Framingham 10 year risk equation (Wilson 1998) will be calculated on all participants with the following data available at baseline and week 48:

- Age
- Gender
- Smoking status
- Fasting total and HDL cholesterol
- Blood pressure
- Personal history of diabetes, hypertension or CVD

Distribution of risk will be described by summarizing the data into these 3 categories: low risk (<10%), moderate risk (10-20%), high risk (>20%) at baseline and at week 48. A Chi-squared test will be used to compare the proportions in CVD risk categories between randomization arms at both baseline and week 48.The null hypothesis is that there is no statistically significant difference between randomization arms in 10 year CVD risk. The change in CVD risk from baseline to week 48, between randomized arms, will be compared by an unpaired t test.

***Objective 2. To estimate and compare changes in the metabolic syndrome equation between LPV/r + 2-3N(t)RTI and LPV/r + raltegravir based ART from baseline to 48 weeks.***

The metabolic syndrome (Alberti, 2006) will be calculated on all participants with the required data available at baseline and week 48.

- Waist circumference
- Triglycerides
- HDL cholesterol
- Blood pressure
- Glucose

The results will be summarised as the number (and %) of participants with the syndrome in each treatment group at baseline and week 48. The null hypothesis is that there is no statistically significant difference between randomization arms in newly acquired metabolic syndrome at week 48. A Chi-squared test will be used to compare the proportion of participants with newly acquired metabolic syndrome at week 48.

***Objective 3. To relate changes in limb fat mass to baseline clinical demographics and ART.***

The association between changes in limb fat mass and ART and clinical demographics (Table 1) from baseline to week 48 will be assessed using linear regression. Any characteristic with a p value< 0.1 in univariate analysis will be assessed for inclusion in a multivariate model (however age, gender and ethnicity will remain in the multivariate model regardless of the univariate results – this is due to the confounding influence of these parameters). Backward stepwise methods will be used to build the multivariate model.

**Table 1. Variables to be tested for association with changes in limb fat mass from baseline to W48**

| **Baseline Variable** | **Description** |
| --- | --- |
| Randomised treatment arm | Categorical |
| Duration of ART (time since first ART) | Continuous |
| Use of thymidine NRTI vs non-thymidine NRTI | Categorical |
| Duration of thymidine NRTI use | Continuous |
| BMI | Continuous |
| Age | Continuous |
| Gender | Categorical |
| Ethnicity | Categorical (as per eCRF) |
| Smoking | Categorical (as per eCRF) |
| Blood Pressure | Continuous |
| Lipids (chol, HDL, LDL, trigs, chol:HDL ratio) | Continuous |
| Glycaemic markers (BSL, HOMA, insulin) | Continuous |
| Concomitant medication (anti hypertensives, lipid lowering therapy, testosterone supplementation) | Categorical |
| Duration HIV infection | Continuous |
| CDC category | Categorical |
| CD4 T lymphocyte count | Continuous |
| Nadir CD4+ T lymphocyte count | Continuous |
| CD8 T lymphocyte count | Continuous |
| Plasma HIV RNA | Continuous |
| Limb fat | Continuous |
| Lean mass | Continuous |

**6.2 BONE**

***Objective 1. To estimate and compare changes in 10-year risk of major fracture or hip fracture using the FRAX equation between LPV/r + 2-3N(t)RTI and LPV/r + raltegravir based ART from baseline to 48 weeks.***

The 10 year risk of major fracture or hip fracture equation will be calculated on all participants with the required data available at baseline and week 48. The results will be summarised as mean, standard deviation, median, inter-quartile range, minimum and maximum. The distribution of these data will be presented graphically (either by histogram or box plot).

Country specific algorithms will be used for India and Argentina. For South Africa, Thailand and Malaysia (checking with FRAX group).

The null hypothesis is that there is no statistically significant difference between randomization arms in the 10 year risk of major fracture or hip fracture. An unpaired t-test will be used to compare the change in the 10 year fracture risk between randomization arms from baseline to week 48.

***Objective 2. To relate changes in lumbar spine and femur BMD to baseline clinical demographics and ART. These two models will be run separately.***

The association between changes in BMD and baseline body composition, ART and clinical demographics from baseline to week 48 will be assessed using linear regression. Any characteristic with a p value< 0.1 in univariate analysis will be assessed for inclusion in a multivariate model (however age, gender and ethnicity will remain in the multivariate model regardless of the univariate results – this is due to the confounding influence of these parameters). Forward stepwise methods will be used to build the multivariate model.

**Table 2. Variables to be tested for association with percent change in BMD from baseline to W48**

| **Baseline Variable** | **Description** |
| --- | --- |
| Randomised treatment arm | Categorical |
| Duration of ART (time since first ART) | Continuous |
| Use of tenofovir vs no tenofovir | Categorical |
| Duration of TDF use | Continuous |
| BMI | Continuous |
| Age | Continuous |
| Gender | Categorical |
| Ethnicity | Categorical (as per eCRF) |
| Smoking | Categorical (current, recent, past, never) |
| Blood Pressure | Continuous (systolic and diastolic) |
| Duration HIV infection | Continuous |
| CDC category | Categorical |
| CD4 T lymphocyte count | Continuous |
| CD8 T lymphocyte count | Continuous |
| Plasma HIV RNA | Continuous |
| Baseline peripheral and total body fat mass | Quartiles |
| Years of schooling | Continuous |
| Employment status | Categorical (as per CRF) |
| Monthly income | Categorical (as per CRF) |
| Number of dependents | Categorical (as per CRF) |
| Previous fractures | Categorical (as per CRF) |
| Prior hypogonadism | Categorical (as per CRF) |
| Prior corticosteroids or recreational drugs (any) | Categorical (as per CRF) |
| Alcohol use | Categorical (2 drinks per day or <, >2 drinks per day) |
| Family history of hip fracture | Categorical (as per CRF) |
| Physical activity (walking, moderate, vigorous) | Continuous |
| Absolute and % total body fat. | Continuous |
| Absolute and % total lean mass. | Continuous |
